# Astrocytes rescue neuronal health after cisplatin treatment through mitochondrial transfer

**DOI:** 10.1186/s40478-020-00897-7

**Published:** 2020-03-20

**Authors:** Krystal English, Andrew Shepherd, Ndidi-Ese Uzor, Ronnie Trinh, Annemieke Kavelaars, Cobi J. Heijnen

**Affiliations:** 1 Division of Internal Medicine, Department of Symptom Research, Laboratories of Neuroimmunology, The University of Texas MD Anderson Cancer Center, Houston, TX 77030 USA; 2 Department of Neurobiology & Anatomy, The University of Texas McGovern Medical School, Houston, TX 77030 USA; 3 Department of Neurology, The University of Texas McGovern Medical School, Houston, TX 77030 USA

**Keywords:** Astrocytes, Cortical neurons, Mitochondria, Cisplatin, Miro-1

## Abstract

Neurodegenerative disorders, including chemotherapy-induced cognitive impairment, are associated with neuronal mitochondrial dysfunction. Cisplatin, a commonly used chemotherapeutic, induces neuronal mitochondrial dysfunction in vivo and in vitro. Astrocytes are key players in supporting neuronal development, synaptogenesis, axonal growth, metabolism and, potentially mitochondrial health. We tested the hypothesis that astrocytes transfer healthy mitochondria to neurons after cisplatin treatment to restore neuronal health.

We used an in vitro system in which astrocytes containing mito-mCherry-labeled mitochondria were co-cultured with primary cortical neurons damaged by cisplatin. Culture of primary cortical neurons with cisplatin reduced neuronal survival and depolarized neuronal mitochondrial membrane potential. Cisplatin induced abnormalities in neuronal calcium dynamics that were characterized by increased resting calcium levels, reduced calcium responses to stimulation with KCl, and slower calcium clearance. The same dose of cisplatin that caused neuronal damage did not affect astrocyte survival or astrocytic mitochondrial respiration. Co-culture of cisplatin-treated neurons with astrocytes increased neuronal survival, restored neuronal mitochondrial membrane potential, and normalized neuronal calcium dynamics especially in neurons that had received mitochondria from astrocytes which underlines the importance of mitochondrial transfer. These beneficial effects of astrocytes were associated with transfer of mitochondria from astrocytes to cisplatin-treated neurons. We show that siRNA-mediated knockdown of the Rho-GTPase Miro-1 in astrocytes reduced mitochondrial transfer from astrocytes to neurons and prevented the normalization of neuronal calcium dynamics.

In conclusion, we showed that transfer of mitochondria from astrocytes to neurons rescues neurons from the damage induced by cisplatin treatment. Astrocytes are far more resistant to cisplatin than cortical neurons. We propose that transfer of functional mitochondria from astrocytes to neurons is an important repair mechanism to protect the vulnerable cortical neurons against the toxic effects of cisplatin.

## Significance statement

Chemotherapy-induced neurotoxicity is a serious health problem and little is known about the underlying mechanisms. Especially neurons are very sensitive to cisplatin treatment while astrocytes are not. We show that astrocytes can protect neurons damaged by cisplatin by improving neuronal survival, mitochondrial health, and calcium dynamics in vitro. This beneficial effect of astrocytes is dependent on the transfer of mitochondria from astrocytes to the damaged neurons. Our findings provide evidence for an important endogenous protective neuro-glial mechanism that could contribute to prevention of neuronal death as a result of cisplatin treatment and thereby aid in sustaining brain health of patients during chemotherapy.

## Introduction

Mitochondria are unique organelles that are crucial for sustaining cellular health through multiple functions, including ATP production via oxidative phosphorylation, metabolic regulation, regulation of apoptosis, and Ca^2+^ buffering [1–3]. Neurons are highly specialized cells that, like all cells, are critically dependent on intact mitochondrial function for rapidly responding to changes in energy demand, storing and buffering Ca^2+^ and, specifically for neurons, neurotransmission and plasticity [4–7]. Due to the critical importance of mitochondria for multiple key aspects of neuronal function, it is not surprising that mitochondrial dysfunction can have devastating effects on brain function [8–10].

Although cisplatin treatment of neurons in vitro or ex vivo leads to cell death, adult neurons do not massively die after cisplatin treatment in vivo suggesting there is an endogenous protective mechanism in place [8]. However, long lasting cisplatin treatment leads to neuronal mitochondrial dysfunction, which has severe consequences for brain function including cognition [8, 11].

Astrocytes release multiple factors that are essential to neuronal development, signaling, metabolism, axonal growth and synaptogenesis [12–16]. Recent evidence indicates an additional way via which astrocytes can contribute to neuronal health is by donating healthy mitochondria to damaged neurons [17, 18]. Specifically, Wang et al. showed that exposure of rat hippocampal astrocytes and neurons to H_2_O_2_ or serum deprivation promotes transfer of mitochondria from astrocytes to neurons. Moreover, astrocytes not only function as donor of healthy mitochondria but can also function as recipient of damaged mitochondria. Davis et al. showed that retinal ganglion cell axons routinely shed mitochondria at the optic nerve head to be degraded by astrocytes in vivo. Mitochondrial transfer from one cell type to another is not exclusive to astrocytes and neurons [19]. For example, we and others have shown that mesenchymal stem cells transfer mitochondria to damaged neuronal stem cells thereby improving stem cell survival and mitochondrial membrane potential in the recipient cells [20–22]. Transfer of mitochondria from one cell to another occurs via multiple mechanisms such as release and uptake of vesicles, transfer via gap junctions, and transfer via F-actin based tunneling nanotubes [17, 23]. Mitochondrial Rho-GTPase 1 (Miro-1) is a calcium-sensitive adaptor protein that drives movement of mitochondria along microtubules [24–28]. Miro-1 is involved in transferring mitochondria from mesenchymal stem cells to neuronal stem cells [20, 29], but its contribution to mitochondrial transfer from astrocytes to neurons is unknown.

Multiple neurodegenerative disorders, including Parkinson’s disease, Alzheimer’s disease, and chemotherapy-induced cognitive impairment are associated with neuronal mitochondrial dysfunction [2, 8–10, 30–32]. We have shown recently that treatment of mice with the chemotherapeutic drug cisplatin results in synaptosomal mitochondrial dysfunction that causes cognitive deficits [8]. Cisplatin crosses the blood-brain barrier at levels that are sufficient to cause damage to hippocampal neurons and to neuronal stem cells [33]. However, cisplatin treatment in vivo does not lead to overt neuronal cell death which could indicate that there are endogenous protective mechanisms, such as mitochondrial transfer by astrocytes, to assist in sustaining neuronal health in conditions of acute danger to adult neurons.

The aim of the current study is to test the hypothesis that astrocytes transfer mitochondria to neurons damaged by cisplatin and thereby improve neuronal function and health in vitro.

## Materials and methods

### Culture of cortical neuron and astrocytes

Timed-pregnant Long Evans rats (Charles River, Wilmington, MA, USA) were sacrificed and E18 fetuses of both sexes were collected in accordance with Institutional Animal Care and Use Committee-approved protocols.

Cortices were dissected and incubated in 10 ml of dissociation media (81.8 mM Na_2_So_4_, 30 mM K_2_SO_4_, 5.8 mM MgCl_2_, 0.25 mM CaCl_2_, 1 mM Hepes, 20 mM glucose, 0.0001% Phenol Red, 0.16 mM NaOH pH = 7.4) that contained 10 U/mL papain and 5 mg of L-cysteine in total (Worthington, Lakewood, NJ, USA) for 10 min at 37 °C, followed by incubation with 150 mg of trypsin inhibitor (Millipore-Sigma, St. Louis, MO) in 10 ml of dissociation media for 10 min at 37 °C. The cortical tissue was mechanically dissociation in Opti-mem (GIBCO, Carlsbad, CA, USA) with 2.5 M Glucose (GIBCO). Cells were cultured on plates coated with 0.05 mg/ml poly-D-lysine (PDL; Millipore-Sigma) in neurobasal medium (NBM) with 100 U/mL penicillin and 1x B-27 supplement (Invitrogen Carlsbad, CA) at 37 °C and 5% CO_2_. Neuronal cultures were maintained in NBM with B-27 supplement and media was replaced every 3 days. Cortical astrocytes were grown in DMEM/F12 medium supplemented with 10% fetal bovine serum and 5% of 10,000 units/ ml of penicillin and 10,0000 μg/ml of streptomycin (GIBCO) at 5% CO_2_ and 37 °C.

Neuronal cells were used for experiments after 12–15 days of culturing in vitro (DIV). To confirm neuronal enrichment, cells were fixed with 4% paraformaldehyde in PBS, treated with 0.25% Triton X-100, blocked in 2% BSA in PBS and stained with anti-Map2 antibody (1:2000); Sigma-Aldrich); anti-GFAP (1:200, Acris, Rockville, MD) and anti-Olig2 (1:400, Abcam, Cambridge, UK) antibody. On DIV12 > 98% of cells were Map2+ and Olig2 and GFAP-negative. Astrocytes were used until the third passage and were > 99% GFAP+.

### Astrocyte transfections

Astrocytes were plated in a 6-well plate at 1.5 × 10^5^ cells/well 24 h before transfection. For labeling mitochondria, astrocytes were transfected with 2 μg of pLYS1-FLAG-MitoGFP-HA (Addgene plasmid # 50057) which contains the pore-forming subunit of the mitochondrial calcium uniporter coupled to GFP or a mito-mCherry construct generated by subcloning the targeting sequence of the pLYS1-FLAG-MitoGFP-HA plasmid into the mcherry2-N1 vector (Addgene plasmid # 54517). For Miro-1 knockdown, 5 nmol of Rho-1 siRNA (Qiagen, Germany #SI01401743) was diluted in rnase-free water (provided in kit) to make a 20 μM stock, andAllStars Negative Control Scrambled siRNA (Qiagen #SI03650318) was performed similarly to make a 20 μM stock instructions provided. Astrocytes were transfected with 80 nM of Rho-1 siRNA from a 20 μM stock (Qiagen, Germany #SI01401743) or 80 nM AllStars Negative Control Scrambled siRNA from a 20 μM stock (Qiagen #SI03650318). All transfections were performed using the Astrocyte Transfection kit (Altogen Biosystems, Las Vegas, NV, USA) according to manufacturer’s instructions. Miro-1 knockdown was confirmed by Western blot with anti-Rho1 antibody (Novus Biologicals, Centennial, CO, USA) with GAPDH as control (Abcam) followed detection of bands with enhanced chemoluminescence (GE Healthcare Bio-Sciences, Pittsburgh, PA). Blots were captured in the LAS system using Image Quant software (GE Healthcare Bio-Sciences) for quantification of bands.

### Analysis of neuronal survival

Neurons were plated in 96 well plates coated with 0.05 mg/ml PDL at 5 × 10^4^ neurons/well. Viability after exposure to cisplatin (Teva, Petah Tikva, Israel) was quantified using the colorimetric cell viability reagent WST-1 (Millipore-Sigma, #11644807001). To assess the effect of astrocytes on neuronal survival, separate cultures of neurons (1.5 × 10^5^ cells/well in a 6-well plate) and astrocytes (5 × 10^4^ /well in a 6-well plate) were treated with cisplatin for 24 h. Neurons were then labeled with 20 μM CellTracker Blue (CTB; Invitrogen) for 45 min at 37 °C, and washed in serum-free media. Astrocytes (5 × 10^4^ cells/well) were added to the neuronal culture and survival of CTB+ neurons was quantified 17 h later using a Countess II FL automated cell counter (Invitrogen).

### Analysis of mitochondrial membrane potential and mitochondrial transfer

Neurons were plated at 1.5 × 10^5^ cells/well in a 6-well plate, treated with cisplatin for 24 h, and labeled with 20 μM CellTracker Green (Thermo Fisher). Astrocytes (5 × 10^4^ cells/well) were added and co-cultured with the neurons for 17 h. The co-cultures were stained with tetramethylrhodamine methyl ester (TMRM, Invitrogen; 250 nM) for 45 min at 37 °C, or Mitotracker (50 nM, Thermo Fisher, Waltham, MA, USA) for 30 min at 37 °C. As a positive control, neurons were treated with 10 μM carbonilcyanide p-triflouromethoxyphenylhydrazone (FCCP, Sigma-Aldrich), a mitochondrial uncoupler, for 15 min. Cells were collected and TMRM fluorescence intensity of the cell tracker green positive cells was quantified using an Accuri C6 Flow Cytometer (BD Biosciences, San Jose, CA USA). For confocal microscopy, neurons were plated in cell culture imaging dishes (ibidi, Fitchburg, WI, USA), treated with cisplatin, stained with CTB and cultured with or without mito-GFP and cell tracker deep red-labeled astrocytes for 17 h followed by staining with TMRM or Mitotracker. The TMRM was used in sub-quench mode as described previously [34].

For analysis of mitochondrial transfer, neurons exposed to cisplatin or vehicle were labeled with 20 μM CTB (Thermo Fisher) prior to co-culture with astrocytes. Astrocytes were transfected with either mito-GFP or mito-mCherry prior to exposure to cisplatin followed by labeling with 20 μM Celltracker DeepRed (Thermo Fisher) and co-culture with neurons. Neurons containing an area positive for the mito-GFP or mito-mCherry signal that was larger than 8 pixels were scored as containing astrocyte-derived mitohcondria. The co-cultures were imaged on an SPE Leica Confocal Microscope (Leica Microsystems, Buffalo Grove, IL, USA) with a 63 X or 40 X objective and images were analyzed with LAS X software.

### Analysis of mitochondrial bioenergetics

To assess mitochondrial bioenergetics, astrocytes (5 × 10^4^ cells/well) were plated in a Seahorse XFe 24 microplate (Seahorse Biosciences/Agilent Technologies, Santa Clara, CA, USA) coated with 0.05 mg/ml PDL and treated with 1 μM cisplatin or vehicle for 24 h. Cells were washed and incubated for 1 h at 37 °C in XF base media (Seahorse Biosciences) supplemented with 11 mM glucose (Sigma-Aldrich), 2 mM glutamine (Sigma Aldrich), and 1 mM pyruvate (Sigma-Aldrich), 2 mM Oligomycin (Sigma-Alrich), 4 mM FCCP, and rotenone/antimycin A (Sigma-Aldrich, 2 mM each) were used with a 3-time repeat of a 2-min mix, 3-min wait, and 2-min measure assay cycle. Oxygen consumption rates were normalized to the total protein content of each well. Basal respiration, maximal respiratory capacity, and spare respiratory capacity were determined as described previously [20].

### Calcium imaging

Functional Ca^2+^ imaging on cortical neurons was performed as described previously [35]. 12 mm circular glass coverslips containing cells were incubated at room temperature for 20 min with the Ca^2+^-sensitive dye Fura-2-AM (Invitrogen, 2 μM), dissolved in standard extracellular HEPES-buffered HBSS (known hereafter as extracellular imaging buffer) containing the following (in mM): 140 NaCl, 5 KCl, 1.3 CaCl_2_, 0.4 MgSO_4_, 0.5 MgCl_2_, 0.4 KH_2_PO_4_, 0.6 NaHPO_4_, 3 NaHCO_3_, 10 glucose, and 10 HEPES adjusted to pH 7.4 with NaOH and 310 mOsm with sucrose. The coverslip was placed in the recording chamber (ALA scientific Instruments, Farmingdale, NY, USA) mounted on the stage of an inverted Nikon Ti2 microscope and continuously superfused for 5 min at room temperature with extracellular imaging buffer. Fura-2 fluorescence was alternately excited at 340 and 380 nm (12 nm band-pass, 50 ms exposure) at 1 Hz using a Lambda LS Xenon lamp (Sutter Instruments, Novato, CA, USA) and a 10x/NA 0.5 objective or 40x/NA 0.6. The CFI Super Fluor 10X was used to measure the ratio of Fura-2 fluorescence. Emitted fluorescence was collected at 510 nm using a sCMOS pco.edge camera for the entire experimental duration, including the first 5 min wash duration, and the ratio of fluorescence (F340:F380) was calculated. The shift in the ratio of Fura-2 fluorescence between excitation at 340 nm versus 380 nm is used as a readout of changes in intracellular calcium concentration [Ca^2+^_i_]. Baseline recording of F340:F380 ratio without stimulus was completed to obtain an indication of resting [Ca^2+^_i_]. Neurons were stimulated with 20 mM of KCl in the extracellular imaging buffer with continuous superfusion at room temperature. Imaging was analyzed with the NIS Elements software. To identify neurons that did or did not receive astrocytic mitochondria, the Nikon inverted Ti2 microscope with associated A1Rsi-HD confocal system and NIS Elements software was used with the CFI S Plan Fluor ELWD 40XC objective. A single exposure at 576 nm excitation was used to quantify fluorescence within neurons before beginning measurement of F340:F380 ratio.

### Data analysis

Data are presented as mean ± SEM of at least 3 independent experiments performed in duplicate or triplicate. For survival analysis and mitochondrial membrane potential analysis, data were normalized to the control of each experiment and replicates were averaged. We used One-way or Two-way analysis of variance (ANOVA) with or without repeated measure followed by Tukey’s correction for multiple comparisons or Sidak’s correction for multiple comparisons according to experimental set up. For mitochondrial transfer analysis, the percentage of neurons that received mitochondria was calculate for each group. We used Student’s t-test or Two-way analysis of variance (ANOVA) without repeated measures followed by Tukey’s correction for multiple comparisons. For calcium imaging, the ratio of fluorescence (*F340:F380*) was calculated and the ratios at baseline were subtracted from the maximum peak values upon KCl stimulus to calculate change in [Ca^2+^_i_]. To calculate Ca^2+^ clearance, *F340:F380 ratios were converted to a 0–1 scale with 1 being the maximum value in response to 20 mM KCl. Then, we quantified the time to return to 20% of baseline by counting the values from 1 (maximum peak) to 20% of the baseline value for each neurons.* All analyses were performed using GraphPad Prism 8 (GraphPad Software, La Jolla, CA, USA).

## Results

### Astrocytes improve neuronal survival after cisplatin treatment

**Fig. 1:**
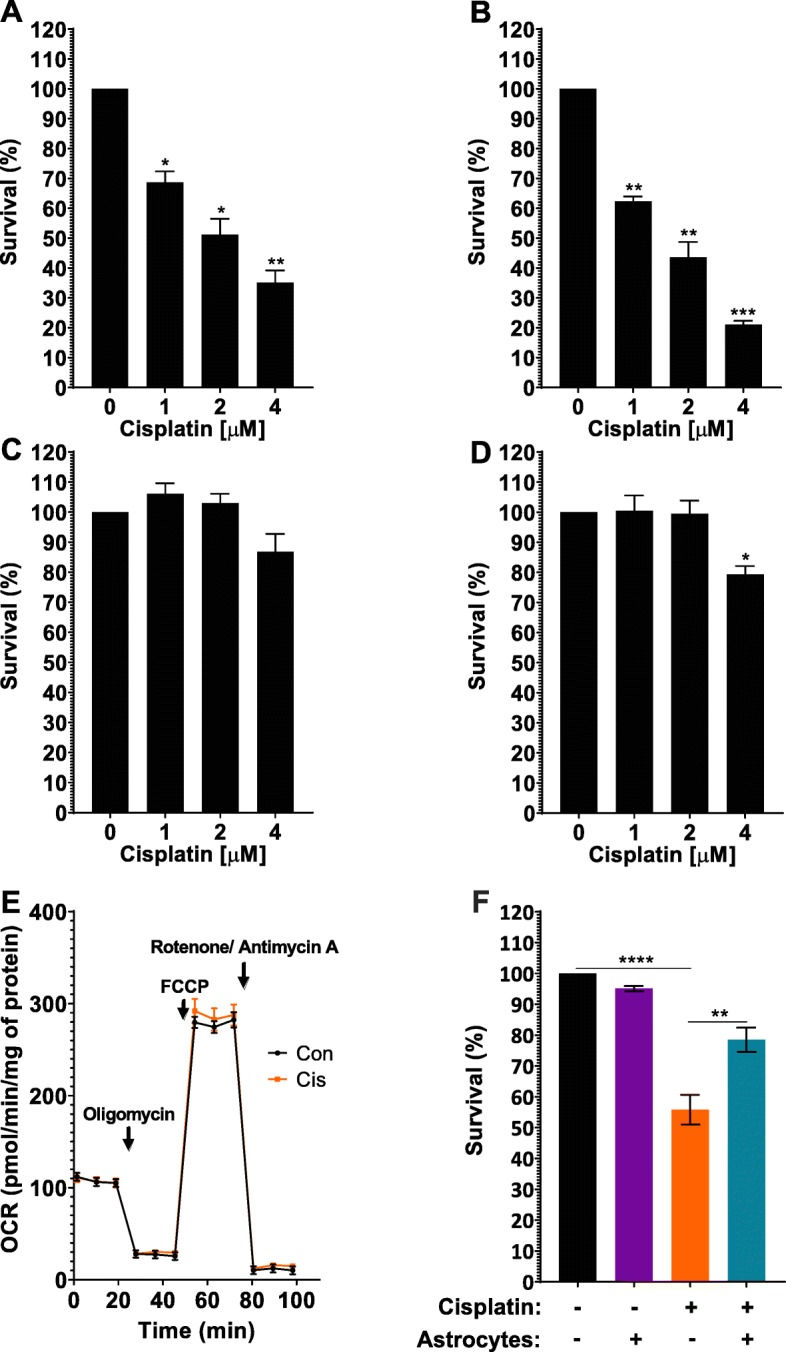
Astrocytes improve survival of neurons damaged by cisplatin. **a**-**d**. Survival of neurons (**a**, **b**) and astrocytes (**c**, **d**) after exposure to cisplatin. Primary cultures of cortical neurons (12 days in vitro) or cortical astrocytes were treated with increasing doses of cisplatin or vehicle for 24 h, then the cisplatin was removed and replaced with media. Survival was measured using a WST-1 assay: (**a**, **c**) at the end of the 24 h culture period or (**b**, **d**) 17 h after removal of cisplatin. Data was normalized to survival in the absence of cisplatin and analyzed using one-way ANOVA followed by Dunnett’s multiple comparisons test (*p < 0.05, **p < 0.01, ***p < 0.001). **e**. Astrocytes were treated with 1 μM cisplatin for 24 h followed by 17 h of culture without cisplatin. Oxygen consumption rates (OCR) were analyzed using Seahorse XFe 24 Analyzer and normalized to protein content. **f**. Neurons and astrocytes were treated with cisplatin or control medium for 24 h. Cisplatin was removed, neurons were labeled with Celltracker Blue (CTB) and cultured for another 17 h with or without astrocytes, and the number of surviving neurons was counted. Data are normalized to control and represents the mean ± SEM of 3 independent experiments performed in duplicate. Two-way ANOVA: cisplatin x astrocyte interaction: **p < 0.01; Tukey’s multiple comparisons post hoc test (*p < 0.05, **p < 0.01, ****p < 0.0001)

To examine the effect of cisplatin on neuron and astrocyte survival, we treated separate cultures of primary cortical neurons and astrocytes with cisplatin for 24 h and measured survival using the WST assay. Cisplatin dose-dependently reduced neuronal survival as assessed immediately after removal of cisplatin (Fig. 1a) or 17 h later (Fig. 1b). Astrocytes were shown to be resistant to cisplatin since only the highest dose (4 μM) resulted in a modest reduction in astrocyte survival (Fig. 1c and d). For all further experiments we used cisplatin at a dose of 1 μM. Exposure of astrocytes to 1 μM cisplatin did not induce detectable changes in astrocytic mitochondrial respiration; basal respiration, spare respiratory capacity and maximal respiration were similar in control and cisplatin-treated astrocytes (Fig. 1e).

To test the hypothesis that astrocytes improve the survival of neurons treated with cisplatin, primary cortical neurons and astrocytes were cultured separately in the presence or absence of 1 μM cisplatin for 24 h. Next cisplatin was removed, neurons were labeled with celltracker blue (CTB), and co-cultured with astrocytes for an additional 17 h. Co-culture with astrocytes significantly improved survival of cisplatin-treated neurons. Astrocytes did not affect survival of control neurons (Fig. 1f).

### Astrocytes improve mitochondrial membrane potential of neurons damaged by cisplatin

**Fig. 2:**
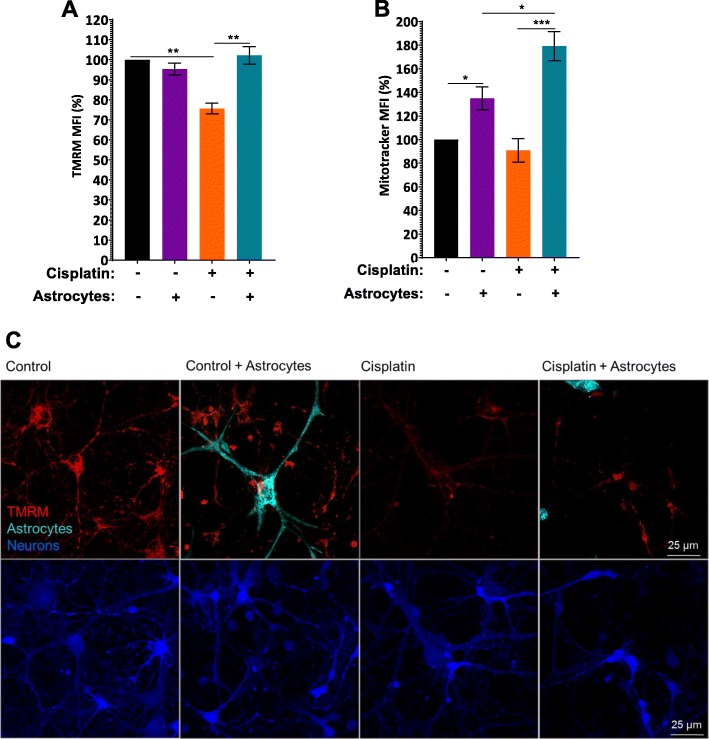
Astrocytes improve mitochondrial membrane potential of neurons damaged by cisplatin. After treating neurons and astrocytes separately with and without cisplatin for 24 h, neurons were labeled with Celltracker Green (CTG) and co-cultured with astrocytes for 17 h. Cells were labeled with TMRM (**a**) to assess mitochondrial membrane potential or with mitotracker (**b**) to quantify mitochondrial content by FACS analysis. Fluorescence intensity was normalized to fluorescence intensity in control CTG-positive neurons for each experiment and data represents the mean ± SEM of 3 independent experiments performed in duplicate. Two-way ANOVA TMRM: cisplatin x astrocyte interaction: *p < 0.05; Tukey’s post-hoc test: **p < 0.01. Mitotracker: cisplatin x astrocyte interaction: *p < 0.05; Tukey post-test: *p < 0.05, ***p < 0.001. **c**. Representative confocal images of the TMRM signal (red) with neurons labeled with CTB and astrocytes with cell tracker deep red (Teal). Scale bar: 25 μm

To assess the effect of cisplatin on neuronal mitochondrial integrity, we quantified mitochondrial membrane potential by labeling the cells with tetramethylrhodamine (TMRM) and assessed fluorescence intensity by flow cytometry. Figure 2a clearly shows that cisplatin depolarized the neuronal mitochondrial membrane potential as indicated by a reduction in TMRM fluorescence intensity. The cisplatin-induced reduction in TMRM fluorescence intensity was not associated with changes in neuronal mitochondrial content as determined by labeling mitochondria with the membrane potential-independent dye Mitotracker (Fig. 2b). Together these findings indicate that cisplatin reduces neuronal mitochondrial membrane potential without leading to an actual loss of mitochondria under the conditions tested. Co-culture with astrocytes restored the mitochondrial membrane potential of neurons cultured with cisplatin (Fig. 2a). Confocal microscopy confirmed that cisplatin reduces TMRM staining in somata and dendrites of neurons and that co-culture with astrocytes restored TMRM staining (Fig. 2c). The increase in TMRM fluorescence intensity of cisplatin-treated neurons in response to astrocytes was associated with an increase in mitotracker fluorescence intensity in neuronal cells (Fig. 2b), indicating an increase in neuronal mitochondrial content as a result of co-culture with astrocytes.

### Astrocytes transfer mitochondria to neurons damaged by cisplatin

**Fig. 3:**
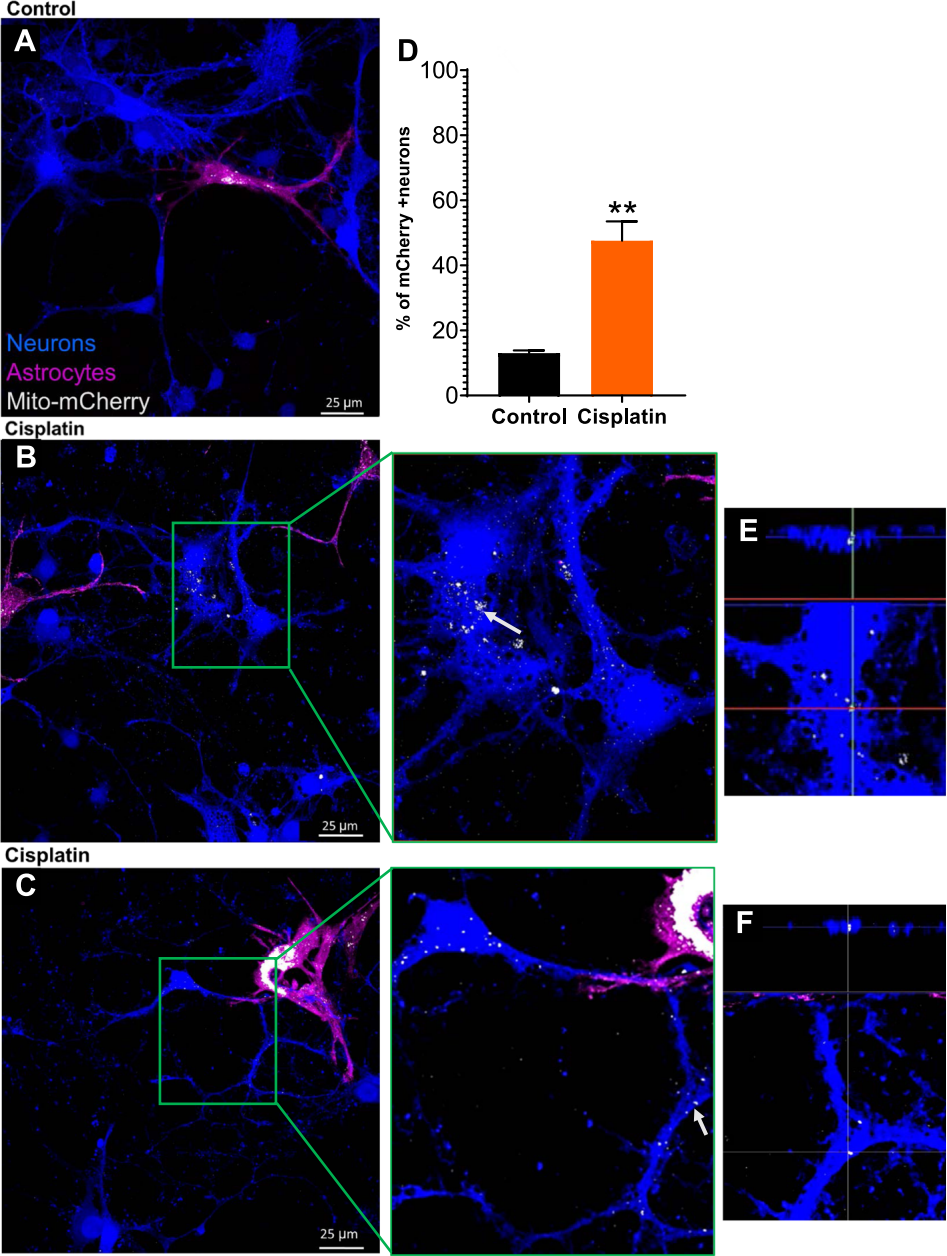
Astrocytes transfer mitochondria to neurons damaged by cisplatin. **a**. Representative confocal image of untreated neurons co-cultured for 17 h with astrocytes in which mitochondria were labeled with mCherry. **b**-**c**. Representative confocal images of co-cultures of cisplatin-treated neurons labeled with CTB and cisplatin-treated astrocytes in which mitochondria were labeled with mCherry. Middle panel: larger magnification of the boxed area in B/C. **e**-**f**. 3-D reconstruction and orthogonal slicing showing that the astrocyte-derived mCherry-positive mitochondria (identified by arrows in the middle panel) are present within the neurons. Scale bar: 25 μm. **d**. Quantification of neurons in co-cultures containing mCherry+ astrocyte-derived mitochondria. Paired Student’s t-test **p < 0.01. Data represents the mean ± SEM of 3 independent experiments performed in duplicate

Next, we tested the hypothesis that astrocytes transfer mitochondria to neurons damaged by cisplatin. We labeled astrocyte mitochondria with mCherry coupled to a mitochondrial localization sequence (mito-mCherry). Neurons and astrocytes were treated separately with cisplatin or control medium for 24 h; neurons were labeled with Celltracker Blue, and co-cultured with Celltracker Deep Red-labeled astrocytes for an additional 17 h. Confocal fluorescence analysis revealed that cisplatin-treated neurons contained astrocyte-derived mCherry+ mitochondria. Only a few astrocyte-derived mitochondria were present in untreated control neurons co-cultured with astrocytes (Fig. 3a-c). These findings indicate that astrocytes transfer mitochondria to neurons damaged by cisplatin. Quantitative assessment of mitochondrial transfer showed that cisplatin induced an approximately 3-fold increase in the percentage of neurons that had received mitochondria from astrocytes (Fig. 3d). 3D reconstruction and orthogonal section analysis confirmed that the mito-mCherry+ mitochondria are localized inside the neurons and were detected in the soma, axons and dendrites of the neuron (Fig. 3e-f).

### Role of Miro-1 in mitochondrial transfer from astrocytes to neurons

**Fig. 4:**
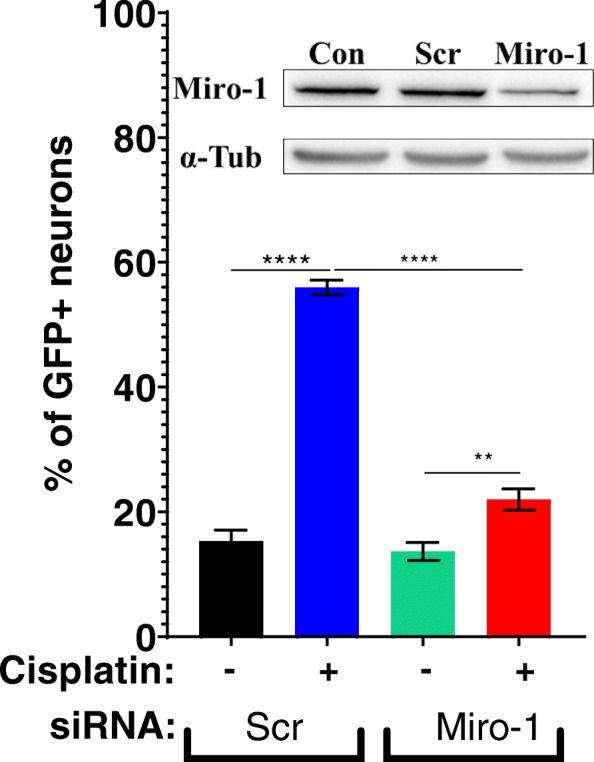
Role of Miro-1 in mitochondrial transfer from astrocytes to neurons. Astrocytes were transfected with mito-GFP and Miro-1 siRNA or control scrambled (Scr) siRNA cultured with or without cisplatin 24 h and co-cultured for 17 h with neurons treated with or without cisplatin. Using confocal microscopy the percentage of neurons containing GFP was quantified. Data represents the mean ± SEM of 3 independent experiments performed in duplicate. Inset: Western blot to confirm knockdown of Miro-1 in astrocytes after transfecting with mito-GFP, Scr and Miro-1 siRNA. The percentage of knockdown was normalized to the control and quantified. Two-way ANOVA cisplatin x transfected astrocyte interaction: ****p < 0.0001, followed by Tukey’s post-hoc test: **p < 0.01, ****p < 0.0001

We next assessed the contribution of Miro-1, a Rho-GTPase mitochondrial adaptor protein, to mitochondrial transfer from astrocytes to damaged neurons. Astrocytes were transfected with short interfering RNA (siRNA) to knock down Miro-1 (astrocytes^Miro-1 siRNA^) and mitochondria in astrocytes were labeled with mito-GFP. Miro-1 siRNA decreased astrocytic Miro-1 by approximately ~ 50% (Fig. 4 inset). Neurons were treated with cisplatin and co-cultured with astrocytes^Miro-1 siRNA^ or astrocytes^Scr siRNA^ as a control and mitochondrial transfer was quantified. The results in Fig. 4 show that knockdown of Miro-1 in astrocytes decreased mitochondrial transfer to neurons in comparison to astrocytes treated with control scrambled siRNA (Fig. 4). These findings indicate that Miro-1 plays a key role in the transfer of mitochondria from astrocytes to neurons damaged by cisplatin.

### Effects of cisplatin and astrocytes on neuronal calcium dynamics

**Fig. 5:**
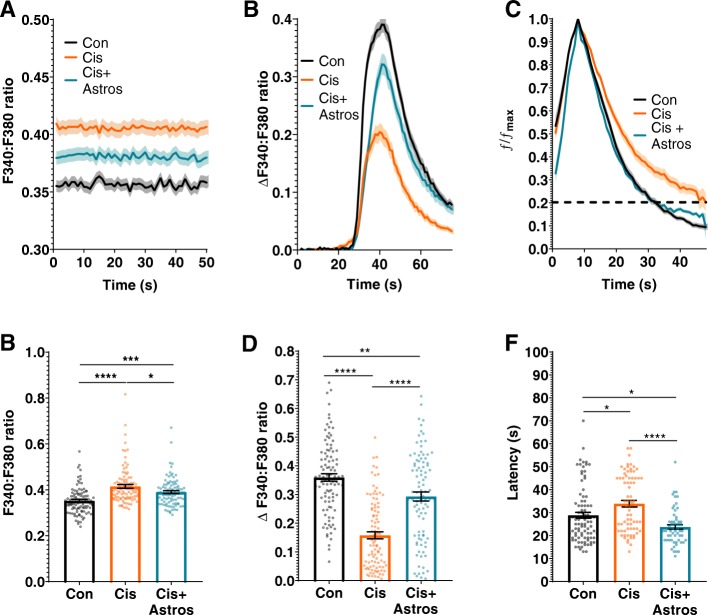
Effects of cisplatin and astrocytes on neuronal calcium dynamics. Neurons were treated with cisplatin followed by co-culture with astrocytes and calcium dynamics were monitored with Fura-2. **a**, **b**. Resting ratio of 340:380 Fura-2 fluorescence intensity (F340:F380) as an indicator of intracellular levels of Ca^2+^ of neurons treated with or without cisplatin followed by co-culture with or without astrocytes. **a**. Mean traces and SEM of the F340:F380 ratio of Fura-2 at baseline during first 50 s of recording. **b**. Mean F340:F380 ratio during 50 s of recording for each neuron at rest. Data represents the mean ± SEM of > 90 neurons total per group collected in 3 independent experiments. One-way ANOVA, followed by Tukey’s post-hoc test: (*p < 0.05, ***p < 0.001, ****p < 0.0001). **c**. Mean traces and SEM of the increase in F340:F380 ratio of Fura-2 of neurons responding to stimulation with 20 mM KCl. The threshold change in Fura-2 F340:F380 ratio to be considered a response to 20 mM KCl was set at an increase of > 5 x standard deviations above baseline average. This excluded 9% of neurons in the control group, 23% of neurons in the cisplatin group and 11% of neurons in the cisplatin + astrocyte group. Data represents the mean ± SEM of 3 independent experiments with > 70 neurons for each group. **d**. Mean change in F340:F380ratio in response to stimulation of neurons with 20 mM KCl. The increase in Fura-2 ratio in response to 20 mM KCl was calculated for each neuron. Data represent the mean ± SEM of *n* > 90 neurons for each group collected in 3 independent experiments. One-way ANOVA, followed by Tukey’s post-hoc test: (**p < 0.01, ***p < 0.001, ****p < 0.0001). **e** Normalized KCl responses show a delayed decay of Ca^2+^ influx in cisplatin-treated neurons vs. control and cisplatin + astrocytes groups. **f**. Quantification of latency to return to 20% of maximal Ca^2+^ (dotted line). Data represents the mean ± SEM of 3 independent experiments with > 70 neurons for each group. One-way ANOVA, followed by Tukey’s post-hoc test: (*p < 0.05, ***p < 0.0001)

We next asked the question whether cisplatin alters neuronal Ca^2+^ levels and whether co-culture with astrocytes affects cisplatin-induced changes in neuronal calcium dynamics. Resting [Ca^2+^_i_] levels, as measured using the calcium indicator Fura-2, were significantly higher in cisplatin-treated neurons than in control neurons (Fig. 5a and b). Co-culture with astrocytes normalized resting [Ca^2+^_i_] levels in cisplatin-treated neurons (Fig. 5a and b). The increase in neuronal [Ca^2+^_i_] level in response to 20 mM KCl was smaller in cisplatin-treated than in control neurons and this was also normalized by co-culture with astrocytes, indicating that astrocytes normalize calcium dynamics in neurons treated with cisplatin. (Fig. 5c and d).

Treatment of neurons with cisplatin increased the time to reach 80% clearance of [Ca^2+^_i_] after exposure to 20 mM KCl (Fig. 5e and f), even though maximal calcium levels were significantly lower in cisplatin-treated neurons (Fig. 5a and b). These findings indicate Ca^2+^ clearance is impaired in cisplatin treated neurons. Co-culture with astrocytes increased the rate of calcium clearance in cisplatin-treated neurons to a level that was even higher than that of control neurons (Fig. 5e and f).

### Mitochondrial transfer underlies the beneficial effects of astrocytes on calcium dynamics in damaged neurons

**Fig. 6:**
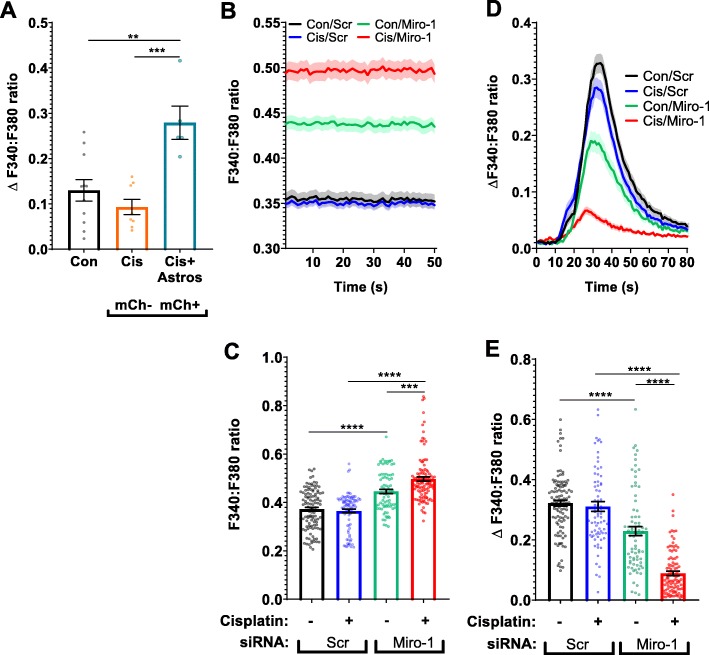
Mitochondrial transfer underlies the beneficial effects of astrocytes on calcium dynamics in neurons damaged by cisplatin. **a**. Neurons were treated with and without cisplatin for 24 h and co-cultured with astrocytes transfected with mito-mCherry (labeled mCh) for 17 h. The calcium response to 20 mM KCl was compared in cisplatin-treated neurons cultured without astrocytes (black bar), or with astrocytes separated into mCherry negative neurons (orange bar; do not contain astrocytic mitochondria) and mCherry positive neurons (blue bar; contain astrocyte-derived mitochondria). The mean increase in the F340:F380 ratio of Fura-2 fluorescence in response to 20 mM KCl was calculated as in Fig. 5. Data represents the mean ± SEM of 3 independent experiments with *n* = 24 neurons in total. One-way ANOVA, followed by Tukey’s post-hoc test: (**p < 0.01, ***p < 0.0001). **b**. Resting F340:F380 ratio of Fura-2 of neurons treated with cisplatin followed by co-culture with astrocytes transfected with mito-GFP and scr siRNA or Miro-1 siRNA. **c**. Mean F340:F380 ratio was calculated for each neuron for first 50 s and data represents the mean ± SEM of 3 independent experiments with *n* > 80 neurons for each group. Two-way ANOVA: cisplatin x transfected astrocyte interaction: ***p < 0.001 followed by Tukey’s post-hoc test: (***p < 0.001, ****p < 0.0001). **d**. Mean traces and SEM of the F340:F380 ratio in response to 20 mM KCl stimulus in neurons treated with and without cisplatin and co-cultured with astrocytes transfected with scrambled (Scr) or Miro-1 siRNA. The mean F340:F380 ratio KCl responses were normalized to baseline. **e**. The change in F340:F380 ratio between baseline and maximum peak value from the 20 mM KCl stimulus was calculated which correlated with [Ca^2+^]_i_ increase in neurons. Mean increase in F340:F380 ratio ratio was calculated for each neuron for 3 s before 20 mM KCl and subtracted from mean F340:F380 ratio at the maximum response to 20 mM KCl. Data represents the mean ± SEM of 3 independent experiments with n > 80 neurons for each group. Two-way ANOVA: cisplatin x transfected astrocyte interaction: (****p < 0.0001), followed by Tukey’s post-hoc test (****p < 0.0001)

To address the question of whether transfer of mitochondria from astrocytes to neurons contributes to the improved neuronal Ca^2+^ dynamics, we compared calcium responses to 20 mM KCl of neurons that did or did not contain mCherry+ mitochondria on the same coverslip. As shown in Fig. 6a neurons that contained astrocyte-derived mitochondria showed a larger increase in 20 mM KCl evoked [Ca^2+^_i_] in comparison to neurons in the same culture that did not receive astrocytic mitochondria (Fig. 6a). This finding indicates that transfer of mitochondria from astrocytes to neurons plays a substantial role in restoring the KCl evoked [Ca^2+^_i_] increase in neurons damaged by cisplatin.

To further address the contribution of mitochondrial transfer to the normalization of calcium dynamics in neurons in response to co-culture with astrocytes, we assessed the effect of astrocytic Miro-1 knockdown. The results in Fig. 6b and c show that basal calcium levels were lower in cisplatin-damaged neurons co-cultured with control astrocytes^Scr siRNA^ as compared to astrocytes^Miro-1 siRNA^. This indicates that knockdown of Miro-1 in astrocytes prevented the normalization of baseline calcium levels in cisplatin-treated neurons (Fig. 6b and c). Similarly, co-culture of cisplatin-treated neurons with astrocytes^Miro-1 siRNA^ failed to normalize the 20 mM KCl evoked [Ca^2+^_i_] that was observed in the presence of control astrocytes^Scr siRNA^ (Fig. 6d and e). This indicates that the restorative effects of astrocytes on calcium dynamics in cisplatin-treated neurons are abrogated by impairing mitochondrial transfer from astrocytes.

## Discussion

Our in vitro findings suggest that astrocytic mitochondrial transfer to neurons may represent an endogenous repair mechanism to counteract the neurotoxic effects of cisplatin treatment. Our evidence indicates that cisplatin reduces neuronal survival and decreases mitochondrial membrane potential in the surviving neurons. We also show that co-culture of cisplatin-treated neurons with astrocytes results in mitochondrial transfer from astrocytes to neurons and this is associated with normalization of survival and mitochondrial membrane potential. Cisplatin altered neuronal Ca^2+^_i_ levels in cortical neurons, and addition of astrocytes normalized neuronal Ca^2+^_i_. Calcium levels were specifically restored in those cisplatin-treated neurons that had received astrocytic mitochondria. Moreover, we show that the Rho-GTPase Miro-1 is essential for the transfer of mitochondria from astrocytes to neurons. SiRNA-mediated knockdown of astrocytic Miro-1 prevented transfer of mitochondria from astrocytes to damaged neurons and prevented the restoration of calcium dynamics in neurons damaged by cisplatin. Collectively, our data support the hypothesis that astrocytes counteract the neurotoxic effects of cisplatin by transfering mitochondria to neurons damaged by cisplatin via a Miro-1-dependent pathway.

The effects of astrocytic mitochondrial transfer have been previously shown in disease models of Alexander’s disease and ischemic stroke [36, 37]. In our in vitro system, we investigated whether astrocytes could also have a restorative effect on neuronal damage as a result of cisplatin treatment. To that end we pre-incubated neurons with cisplatin which caused a significant decrease in neuronal survival and a reduction in the mitochondrial membrane potential in the surviving neurons, and subsequently co-cultured the surviving neurons with astrocytes. Our data show that astrocytes can repair already existing neuronal damage as a result of cisplatin because co-culture with astrocytes restored neuronal mitochondrial membrane potential and protected against further neuronal death. Platinum compounds directly damage the DNA by forming adducts and this interferes with cell proliferation [38]. It is likely that DNA adducts are also formed in neurons exposed to cisplatin, but because these cells are post-mitotic, it is not known what the effect on cell function will be. It should be noted that we have shown previously that in vivo, preventing mitochondrial damage is sufficient to prevent cognitive deficits that develop in response to treatment with cisplatin [8, 39]. Therefore, we propose that mitochondrial toxicity is a key factor in cisplatin-induced damage to neurons.

A structural transport mechanism that has been shown to be involved in intercellular mitochondrial transfer are tunneling nanotubes (TNTs). TNTs are thin non-adherent actin-rich membranous structures with diameters between 50 and 1500 nm that can span long distances of several hundred nm [17, 18, 20, 22, 40–42]. These TNT form direct connections between cells to transport cellular components including cytoplasm, ions, lipid droplets, viral and bacterial pathogens, genetic material and organelles like lysosomes, and last but not least, mitochondria [17, 43, 44]. Multiple authors have described that the mitochondrial Rho-GTPase-1 protein (Miro-1) is a crucial player in intercellular mitochondrial transfer via TNTs. Miro-1 is an outer mitochondrial membrane protein, that binds to Milton, a kinesin/dynein adaptor protein and this promotes mitochondrial motilit y[26, 28, 45, 46]. We have now expanded this knowledge by showing that decreasing Miro-1 expression in astrocytes decreased the transfer of mitochondria from astrocytes to damaged neurons. Apparently, astrocytic Miro-1 is required for transfer of astrocyte mitochondria to neurons. This could imply that the formation of TNTs for intercellular transport is executed by astrocytes rather than neurons. Jiang et al. have described that the cyclic ADP ribose hydrolase CD-38 plays an important role in astrocytic mitochondrial transfer [47]. Inhibition of CD-38 with apigenin significantly reduced astrocytic mitochondrial transfer and it has been suggested that CD-38 may be involved in TNT formation [48]. Furthermore, in an in vitro myeloma cancer model, myeloma cells were shown to receive mitochondria from non-malignant bone marrow stromal cells through CD-38-dependent tumor-derived formation of TNTs [48]. However, we have to keep in mind that this phenomenon could be specific for tumor cells.

Our data shows that astrocytes transfer mitochondria to neurons damaged by cisplatin while there is only very limited transfer under control conditions. Similarly, we have reported previously mesenchymal stem cells transfer more mitohcondria to neuronal stem cells (NSCs) damaged by cisplatin than to control NSCs [20]. Berridge et al. showed that astrocytes transfer more mitochondria to damaged neurons in an ischaemic stroke model in comparison to control [49]. We hypothesize that astrocytes may repond to a “help” signal that is expressed by damaged neurons leading to the transfer of mitochondria. It has been suggested that acculuation of p53 on damaged mitochondrial membranes and/or the release of Damage-Associated Molecular Patterns (DAMPS) could serve as the “help” signal [50–52]. Further studies are needed to identify which signals initiate the transfer process.

Miro-1 modulates mitochondrial shape in response to cytosolic Ca^2+^ stress, a phenomenon which is distinct from fission and fusion [53]. Additionally, Stephen et al. [27], have shown that Miro-1 positions mitochondria in areas within the astrocytic processes that are near neuronal synaptic activity where high energy and Ca^2+^ modulation is necessary. With this in mind it could be possible that astrocytic Miro-1 could be important for positioning mitochondria for transfer in response to neuronal Ca^2+^ changes due to cisplatin damage. It is very well possible that additional activities of astrocytes involved in mitochondrial/cellular health could be of importance as well. However, we specifically observed restoration of [Ca^2+^]_i_ levels in those neurons that actually had received astrocytic mitochondria which implies that mitochondrial transfer plays a crucial role in the restorative effect of astrocytes on neuronal [Ca^2+^_i_] levels and health.

Neuronal Ca^2+^ levels are tightly regulated and are critical for many processes in neurons including neurotransmission, depolarization, and synaptic activities. Mitochondria play an important role in controlling Ca^2+^ levels by taking up, buffering and releasing cytosolic Ca^2+^. Cisplatin can negatively alter Ca^2+^ levels such as has been observed in the dorsal root ganglia [54]. Our findings expand this knowledge by showing that cisplatin leads to dysfunctional cortical neuronal Ca^2+^ levels. Abnormalities in resting and KCl-evoked Ca^2+^ increases and clearance due to treatment of cortical neurons with cisplatin were reversed in the presence of astrocytes. As mitochondria are important for neuronal Ca^2+^ dynamics we suggest that astrocytic mitochondrial transfer is key to normalizing the neuronal mitochondrial network. It remains to be determined whether the normalization of Ca^2+^ dynamics results from a direct role of the donated mitochondria in calcium buffering or a secondary effect on other key regulators of calcium homeostatis such as the endoplasmic reticulum (ER), Golgi apparatus, and peroxisomes, or the functioning of ion channels and pumps [55–59]. However, our findings do show that transfer of mitochondria normalizes neuronal Ca^2+^ dynamics in neurons damaged by cisplatin.

Our cell survival studies showed that astrocytes are much less sensitive to cisplatin than neurons. RNA-seq data [60], show that astrocytes have a higher concentration of the mitochondrial polymerase gamma (polγ) in comparison to neurons which is the sole polymerase involved in mitochondrial replication, mutagenesis and repair of mtDNA. Therefore, we suggest that the releatively high activity of polγ in astrocytes may lead to efficient repair of cisplatin adducts which could contribute to the observed astrocytic resiliency. In addition, astrocytes have a higher concentration of the copper tansporters ATP7a and ATP7b in comparison to neurons and other glial cells [60]. ATP7a and ATP7b are copper transporters that also promote platinum efflux and thereby may contribute to cellular resistance to cisplatin [61–63].

An important translational question still lingers: if astrocytic mitochondrial transfer occurs in the brain after cisplatin treatment, why do patients undergoing chemotherapy still experience neurotoxicity leading to chemotherapy-induced cognitive impairment? One argument could be that the endogenous restorative capacity of astrocytes is no longer sufficient when patients are treated for a long time, which is common for chemotherapy with cisplatin. Indeed, the risk of developing chemobrain increases with duration of treatment [10, 64–67]. Moreover, our preliminary data indicate that exposure of mice to a single round of cisplatin treatment does not induce cognitive deficits, whereas two rounds of cisplatin do induce significant decreases in performance in tests of cognitive function [8, 68]. Although astrocytes may still prevent the actual death of (non self-renewing) adult neurons, they may may fail to completely restore mitochondrial health. The endogenous protective activity of transferring healthy mitochondria from astrocytes to damaged neurons may also become less efficient in the aging brain. From the literature it is known that the severity of the behavioral neurotoxic effects are correlated with age [65, 66, 69]. When endogenous protective mechanisms are not sufficient, interventions aimed at restoring mitochondrial health may provide additional help. Indeed, we showed recently that cell therapy with mesenchymal stem cells or a pharmacolgocial intervention with PFT-μ and HDAC6 inhibitor both reverse cisplatin-induced neuronal mitochondrial abnomarlities as well as cognitive impairment in mice [8, 11, 28, 30, 39, 51, 70].

In conclusion, we propose that astrocytic mitochondrial transfer is an important endogenous protection mechanism against chemotherapy neurotoxicity. Promoting astrocytic mitochondrial transfer could represent interesting therapeutic targets to prevent or treat the devastating effects of chemotherapy on the brain.

## Conclusions

This novel study demonstrates that: 1) Astrocytes rescue survival and function of neurons damaged by cisplatin by donating their mitochondria to the more vulnerable cortical neurons. 2) Mechanistically, we show that cisplatin depolarizes neuronal mitochondrial membrane potential without decreasing neuronal mitochondrial content. Astrocytes restore neuronal mitochondrial membrane potential leading to an increase in mitochondrial content. 3) Miro-1 siRNA decreases mitochondrial transfer from astrocytes to neurons indicating that Miro-1 is involved in astrocytic mitochondrial transfer. 4) Cisplatin increased resting [Ca^2+^_i_] levels in neurons and impaired neuronal calcium clearance. Astrocytes normalized neuronal resting [Ca^2+^_i_] levels and improved neuronal calcium clearance. 5) To investigate that the transfer of mitochondria is crucial for the rescue of neuronal function, we show here that those neurons that contained astrocyte-derived mitochondria had improved response to 20 mM KCl in comparison to neurons that did not contain astrocyte-derived mitochondria. Astrocytes transfected with Miro-1 siRNA, which decreases mitochondrial transfer from astrocytes to neurons, failed to prevent the normalization of baseline calcium levels and response to 20 mM KCl in neurons damaged by cisplatin. In conclusion this data illustrates that astrocytes transfer mitochondria to neurons damaged by cisplatin and restores neuronal calcium dynamics, survival, mitochondrial membrane potential, and increases mitochondrial content. Our findings contribute to further understanding of endogenous protective neuro-glial mechanisms that aid in protection of the brain against the devastating effects of chemotherapy on healthy cells that are vulnerable to cancer treatment.

## Data Availability

The datasets used and/or analyzed during the current study are available from the corresponding author on reasonable request.

## Abbreviations

[Ca^2+^_i_]: Intracellular calcium concentration
Ca^2+^: Calcium
CTB: CellTracker Blue
CTDR: CellTracker Deep Red
CTG: CellTracker Green
F340:F380: ratio of fluorescence at 340 and 380 nm
FCCP: Carbonilcyanide p-triflouromethoxyphenylhydrazone
KCl: Potassium chloride
Miro-1: Mitochondrial Rho GTPase 1, also known as Rho-1
Mito-GFP: GFP coupled to a mitochondrial localization sequence
Mito-mCherry: mCherry coupled to a mitochondrial localization sequence
siRNA: Short interfering RNA
TMRM: Tetramethylrhodamine
TNTs: Tunneling nanotubes
